# Cost of severe hypoglycaemia in patients with type 1 diabetes in Spain and the cost-effectiveness of insulin lispro compared with regular human insulin in preventing severe hypoglycaemia

**DOI:** 10.1111/j.1742-1241.2008.01783.x

**Published:** 2008-07

**Authors:** J Reviriego, R Gomis, J P Marañés, W Ricart, P Hudson, J A Sacristán

**Affiliations:** 1 Medical Department, Lilly S.A.Madrid, Spain; 2 Hospital Clinic i ProvincialBarcelona, Spain; 3 Hospital Clinico UniversitarioSan Carlos, Madrid, Spain; 4 Hospital Josep TruetaGerona, Spain; 5 Independent ConsultantYork, UK

## Abstract

**Objectives:**

To determine the costs of severe hypoglycaemia (SH) in a population of patients with type 1 diabetes mellitus in the Spanish healthcare system and the cost-effectiveness of insulin lispro over regular insulin in preventing SH episodes.

**Methods:**

A retrospective study of 100 patients in three Spanish health centres was performed. Resource utilisation data were collected only for interventions specifically relating to the hypoglycaemic episode. The direct medical costs determined in the analyses were: costs of hospitalisation, diagnostic tests carried out, costs of treatment administered and other associated costs such as visits to the endocrinologist and re-training in glucose control, transportation and assistance of a care-giver. In addition, indirect costs such as days of lost productivity were measured. The incidence rates of SH for insulin lispro and regular insulin were obtained from the literature. The incremental cost-effectiveness of insulin lispro over regular insulin was calculated.

**Results:**

The overall mean cost per episode of SH was €366, comprised of 65.4% direct costs and 35.6% indirect costs. The largest cost was for hospitalisation at €183 per episode. The SH episodes incidence rates for 100 patients per year were 33 and 73 for insulin lispro and 48 (p < 0.05) and 117 (p < 0.01) for regular insulin, in the two clinical trials found in the literature. The additional cost to prevent one episode of SH with insulin lispro over regular insulin ranged from €277 to insulin lispro dominance.

**Conclusions:**

Severe hypoglycaemia has a significant impact on the total cost of diabetes. The use of insulin lispro is associated with reductions in annual costs because of SH and, possibly, the overall effect may be cost neutral or cost saving when total costs are considered. The cost of SH should be included in the analysis of total socio-economic burden of diabetes.

**Disclosures** J. Reviriego and J. Sacristan are currently employees of Lilly Spain. Dr Gomis, Dr Marañes and Dr Ricart have served as consultant and speakers in Lilly medical educational and scientific meetings. In addition all authors at some point have been involved as investigators in Lilly registration trials.

**What's known**

Little or none data were available with respect to the costs of acute diabetes-related complications associated with insulin treatment as is the case for severe hypoglycaemia.

**What's new**

We provide valuable information on the impact of therapeutic alternatives such as the use of an insulin analogue (insulin lispro) vs. regular human insulin on the total cost of diabetes.

## Introduction

Expenses for medical care continue to increase in most countries and the costs of treatment for particular disease have to be weighed against the benefits. Diabetes is a major chronic disease and is known to be associated with significant increases in healthcare expenditure (1,2). In patients with type 1 diabetes the long-term health benefits of improving glycaemic control in reducing the development and progression of complications have been established by interventional long-term studies such as the Diabetes Control and Complications Trial (DCCT) (3). The economic costs associated with the different therapy regimens in such studies have subsequently been evaluated (4). It was shown that the costs of intensive therapy with either multiple daily injections or continuous subcutaneous infusion were more than twice the costs associated with conventional therapy. However, the extra costs could be offset by long-term savings associated with the reductions in complications (5,6).

The DCCT also established that using intensive therapy regimens significantly increased the incidence of hypoglycaemia (7). The rate of severe hypoglycaemia (SH) with intensive therapy was 61.2 events per 100 patient-years compared with a rate for conventional therapy of 18.7 events per 100 patient-years and the increased frequency was sustained over the period of the study. This increase in frequency of SH may be a disincentive to intensive therapy, so more modern insulins with improved time action profiles, such as insulin lispro (Eli Lilly and Company, Indianapolis, IN), a rapid-acting insulin analogue, which can reduce the frequency may encourage patients to follow such regimens. To establish the economic impact of any new treatment all the costs involved must be evaluated. This study was carried out to determine the costs of SH in a population of patients with type 1 diabetes in the Spanish healthcare system and the incremental cost-effectiveness of insulin lispro over regular insulin in preventing SH episodes.

## Patients and methods

This was a retrospective study aimed to review clinical records of 100 type 1 diabetic patients from three Spanish health centres: Hospital Clinic i Provincial, Barcelona (50 patients), Hospital San Carlos, Madrid (25 patients) and Hospital Josep Trueta, Gerona (25 patients). This manuscript only includes aggregated data and summary statistics describing the characteristics of the group (Table 1). Patients were eligible if had experienced at least one episode of SH, defined as any episode that required external assistance, resulted in loss of consciousness or required treatment with glucagon or intravenous (i.v.) glucose, within the 2 years prior to the start of the study. No other specific entry criteria but pregnancy as exclusion criteria, was considered.

**Table 1:** Patients demographics, diabetes duration, treatment and glucose control data at the time of severe hypoglycaemia episodes (*N* = 100)

	Mean ± SD	Range
Age (years)	33.22 ± 12.17	16–61
BMI (kg/m^2^)	23.66 ± 3.01	18.1–32.3
Duration of diabetes (years)	16.9 ± 10.9	0.98–52.8
No insulin injections per day	3.37 ± 1.06	2–6
Mean insulin dose per day (units/kg)	0.72 ± 0.24	0.35–1.60
Time since last change of insulin regimen (months)	17.7 ± 19.3	0.82–130.5
SBGM (number/week) (*n* = 96)	19.1 ± 10.0	2–42
No. of SH last 2 years	2.99 ± 3.82	1–20
BG at the time of the SH episode (mg/dl)	35.54 ± 8.75	17–52
HbA_1c_ at the time of SH (%) (*n* = 46)*	8.12 ± 1.62	5.5–13.7

* HbA_1c_ values were normalised to a 4–6% range. HbA_1c_, glycosated hemoglobin; BMI, body mass index; BG, blood glucose; SBGM, self-blood glucose monitoring; SH, severe hypoglycaemia.

Twenty-two patients of 99 were treated with two injections per day as follows: basal insulins, either neutral protamine hagedorn (NPH) or ultralente (*N* = 9); premixed (*N* = 12); or a combination of a premixed and a basal insulin (*N* = 1). Seventy-seven patients were treated with more than two injections a day of whom 12 were injecting insulin lispro and 65 were injecting regular insulin as rapid-acting insulins. One patient was excluded from this analysis because insulin regimen data was missing. Mean total daily insulin dose was 0.7 ± 0.2 UI/kg.

The data collected for each patient included: demographics, diabetes characteristics, risk factors, self-monitoring of blood glucose and the number of hypoglycaemic episodes in the previous 2 years. For the most recent episode of SH (i.e. their qualifying episode), data were collected for the characteristics of the episode and the resource utilisation during the episodes specifically related to the hypoglycaemic episode.

The direct medical costs included in the analyses were: costs of hospitalisation, diagnostic tests, medication, visits to the endocrinologist and re-training in glucose control by a healthcare provider. Non-medical costs such as transportation and the assistance of a care-giver were included in direct costs. In addition, the indirect costs such as days of lost productivity (defined as inability to work) were estimated and, where the clinical records did not include sufficient information for this, the patients were interviewed during a routine visit or by telephone to obtain the information. Total resource utilisation was calculated as the product of the percentage of patients requiring a particular resource and the number of days involved or the number of diagnostic analyses or medication required. Both direct and indirect costs were calculated as an average per episode from resource utilisation multiplied by the known hospital costs of each resource.

In addition, those estimated cost were used to evaluate the cost-effectiveness of using a rapid-acting insulin analogue, insulin lispro, compared with regular insulin in the treatment of type 1 diabetic patients. Cost-effectiveness was calculated using the above costs and the incidence rates of SH for insulin lispro and regular insulin reported in two randomised, multicentre, 6-month open-label cross-over studies by Anderson et al. (8) and Holleman et al. (9). Those studies were selected because both compare the incidence of SH in patients with type 1 diabetes, treated with insulin lispro or regular human insulin, using the same hypoglycaemia criteria and with similar clinical trial design.

The costs of both treatment (insulin lispro and regular insulin) were calculated adding the cost of the drug to the cost of the episodes of SH in a hypothetic cohort of 100 patients per arm. Effectiveness has been measured as the percentage of SH. Incremental cost-effectiveness ratios [(cost A−cost B)/(effectiveness A−effectiveness B)] were calculated. The cost-effectiveness ratio has been defined as the incremental cost of obtaining one additional unit of health effect when two interventions are compared. Results were reported as monetary units per outcome gained (euros/SH episode avoided).

Data for all resources collected were analysed and reported as mean ± SD. Direct, indirect and total costs were analysed separately. Correlations between costs and variables such as age or number of blood glucose evaluations per week were determined by Pearson's chi-squared test. Differences in costs between gender and with or without loss of consciousness were determined from Wilcoxon significance tests.

Cost data often do not conform to the assumptions for statistical tests comparing differences in arithmetic means. They are usually right-skewed and truncated at zero because of a small number of high-resource use patients, many patients who incur no costs and the impossibility of incurring costs < 0. The most accepted method to compare mean and calculate confidence intervals (CIs) in cost analysis is the non-parametric boostrap method (10). Our data were highly skewed, therefore CIs around the mean were calculated using bootstrapping with 10,000 simulations (11). Direct and indirect costs were given as euros of 2005 (€).

## Results

Fifty-one (51%) clinical records were reviewed from male patients and 49 (49%) from female patients all had type 1 diabetes. Table 1 shows patients demographics, diabetes duration, treatment and glucose control at the time of SH episodes. There was a significant correlation between the number of insulin injections per day and blood glucose monitoring, with patients injecting more than twice per day monitoring glucose 20.5 ± 10.5 times per week compared with patients injecting twice per day monitoring glucose 14.1 ± 6.5 times per week (p = 0.007).

The average time from the qualifying episode of SH to study entry for the whole population was 6.4 months, ranging from 0.03 to 23.3 months and by centre: Hospital Clinic i Provincial (5.4 ± 6.7 months), Hospital San Carlos (7.2 ± 5.9 months) and Hospital Josep Trueta (7.5 ± 6.6 months). There were 73 (73%) patients who were not aware of the hypoglycaemia and who lost consciousness, while 27 patients had awareness and remained conscious. In 75% of cases the patient was assisted by a family member during the episode of SH. Glucagon was administered to 40% of the patients and i.v. glucose was given to 27% for the treatment of hypoglycaemia; no differences between centres were shown.

Average direct, indirect and total costs associated with the episodes of SH are shown in Table 2. The overall mean cost per episode of SH was €366, comprised of 65.4% direct costs and 34.6% indirect costs (i.e. lost productivity).The largest cost was for hospitalisation (€183 per episode), which represented 50% of the total costs. Hospitalisation included visits to the emergency department (35% of the episodes) and inpatient treatment (7% of episodes). Other direct costs comprised 11% of total costs and mainly consisted of follow-up sessions with the endocrinologists, required by 58% of the patients. The data were highly skewed with18% of patients having total costs of < €6.6 each, 43% < €66 and 88% having total costs less than the mean. Results from the bootstrapping analysis showed a 95% CI of €124–380 around the mean direct costs and €211–551 around the mean total cost.

**Table 2:** Mean costs associated with an episode of severe hypoglycaemia, in euros (€), and as a percentage of the estimated total cost

	Costs (€)	% of total
Direct costs	239 (642) [124–380]	65.4
Hospitalisation	183 (615) [74–318]	50.0
Diagnostic analyses	11 (21) [7–15]	2.9
Treatment medications	5 (6) [4–6]	1.4
Other direct costs	41 (52) [31–52]	11.1
Indirect costs	127 (452) [49–235]	34.6
Total costs	366 (863) [211–551]	100.0

Values within parenthesis represent standard deviations and values within square brackets represent 95% CIs.

The total costs varied between each of the three hospitals and the differences were significant (p < 0.001). Therefore, minimum and maximum hospitalisation costs were used with the average resource utilisation to give an estimate of the sensitivity of overall costs. The direct cost of €239 varied from a minimum estimate of €181 to a maximum of €285, while the overall cost of €366 varied from a minimum of €307 to a maximum of €412.

Total costs were not significantly correlated with the age of the patients or with the total incidence of hypoglycaemia. Frequency of SH in the previous 2 years was slightly but significantly correlated (Pearson's r = 0.487; p < 0.001) with total costs. Other factors that significantly influenced costs of the episode of SH are summarised in Table 3. All costs were significantly greater for male patients compared with female patients. Costs were significantly lower for those patients who monitored their blood glucose more than twice per day compared with just twice per day. Loss of consciousness was significantly associated with greater direct costs and total costs.

**Table 3:** Factors that were significantly correlated with the direct, indirect and total costs of an episode of severe hypoglycaemia

Factor	Item	%	Direct costs	Indirect costs	Total costs
Gender	Male	51	300	154	454
	Female	49	176	99	274
	p-value		0.006	< 0.001	< 0.001
Insulin regimen	2 injections/day	22	402	213	615
	> 2 injections/day	77	193	103	296
	p-value		0.052	0.016	0.009
Loss of consciousness	Yes	73	306	154	460
	No	27	60	53	113
	p-value		< 0.001	0.093	0.002
Glucose determinations per week	< 20	46	329	232	561
	≥ 20	50	165	37	201
	p-value		0.034	0.012	0.011

Whereas, the costs analysis was based on the data on qualifying episodes collected in the three study centres as described above, data on episodes of hypoglycaemia from the previous 2 years were available only from two study centres (*N* = 74). In this period, the average number of overall hypoglycaemic episodes was 54.4 (127.7) per patient (median 24 episodes/patient), while the average number of SH episodes (including the qualifying episode) was 2.99 (3.82) per patient (median two episodes/patient). Sixty-one of the 74 patients were on multiple insulin injection regimens (> 2 injections per day). However, there was no statistical correlation between the number of insulin injections per day and the incidence of hypoglycaemia (p = 0.899) or SH (p = 0.378).

### Cost-effectiveness of insulin lispro over regular human insulin

Anderson et al. (8), in his study of 1008 patients, reported 84 episodes when patients using insulin lispro were unable to self-treat during a hypoglycaemic episode and 119 episodes when patients using regular insulin were unable to self-treat: this equals an incidence rate of 33.94 and 47.84 per 100 patients per year for insulin lispro and regular human insulin respectively (p < 0.05). The glycosated hemoglobin (HbA_1c_) levels at end-point were 8.2% in both treatment regimens. Holleman et al. (9), in his study of 199 patients, reported 36 episodes of SH in patients using lispro and 58 in patients using regular insulin: this equals an incidence rate of 73.36 and 116.58 (p < 0.01) per 100 patients per year for lispro and regular insulin respectively. The HbA_1c_ levels were 7.6% for insulin lispro and 7.5% for regular insulin.

Mean drug costs, per patient per year were calculated to be €397 and €308 for insulin lispro and regular insulin groups respectively. Table 4 shows the cost-effectiveness results of applying the two studies of incidence, mean direct and total (direct + indirect) cost, and the upper and lower bounds of the 95% CIs around these means. For example, using the study by Anderson et al., the SH episodes incidence rates for 100 patients per year were 33.94 for insulin lispro and 47.84 for regular insulin (13.9 fewer with insulin lispro). Using the mean total cost of a SH episode (€366), the total cost for 100 patients would be as follows: [(treatment cost A × 100) + (cost of SH × incidence rate of SH for treatment A)]; therefore for insulin lispro it will be [(397 × 100) + (366 × 33.94)] = 52.122 per 100 patients per year. Similarly, for regular insulin the total cost is €48.269 with a cost difference of €3.853 per year with a reduction of 13.9 episodes of SH per 100 treated patients when treated with insulin lispro. Comparing insulin lispro to regular insulin the cost to prevent one episode of SH is therefore 3.853/13.9 = €277 (Table 4).

**Table 4:** Incremental cost-effectiveness of insulin lispro over regular human insulin

	Anderson et al. (8)	Holleman et al. (9)
**Total (direct + indirect) costs used**		
Mean	279.75	Lispro dominant
Lower bound of 95% CI	493.31	16.10
Upper bound of 95% CI	36.15	Lispro dominant
**Direct costs only used**		
Mean	406.34	Lispro dominant
Lower bound of 95% CI	541.86	93.77
Upper bound of 95% CI	271.57	Lispro dominant

Cost to prevent one episode of severe hypoglycaemia: results using mean of the costs and the upper and lower bounds of the 95% confidence intervals (CI) around these cost mean, from two clinical studies.

## Discussion

Intensive insulin therapy to maintain blood glucose levels as close to normal as possible has been shown from several studies to reduce micro- and macrovascular complications of diabetes (3,12,13). However, this increases the likelihood of hypoglycaemia, which is the most frequent and most feared side effect of insulin treatment and is a limiting factor to improving glucose control (14). Further to personal and medical impacts of SH, there is an associated cost impact that has not been explored in detail. The DCCT gave an average cost of a single episode of SH of US$268 (€253 updated to 2005), although this did not take into account any indirect costs (4). Our retrospective analysis in a population of type 1 diabetes patients in Spain, determined that the average overall cost of an episode of SH was €366. This total cost was influenced by various factors such as gender, incidence of loss of consciousness and intensity of the insulin regimen. Indirect costs accounting for 34.6% of the total costs of a severe episode of hypoglycaemia. In the DCCT about a third of the episodes of SH involved loss of consciousness or seizure, regardless of therapy, but the patients were hospitalised for only 5% of these episodes. In the present study 73% of the patients lost consciousness; however, inpatient hospitalisation treatment was only necessary for 7% of the patients. However, this acute complication has a significant impact on cost, as it has been shown in a study of the French population indicating that for the country as a whole, 10,800 hospitalisations per year were due to hypoglycaemia as a total estimated cost of €15–21 million (15).

In our study, the costs were lower for females than for males (Table 3) and for those with a more intensive regimen, injecting insulin more than twice a day, and determining blood glucose ≥ 20 times per week. Indirect costs for females could possibly be accounted for as lower wages. Lower costs in patients using a more intensive regimen may be associated with better glycaemic control and less glucose variation because of closer blood glucose monitoring. Similar data on costs and utilisation between users of insulin lispro, a human insulin analogue with an improved action profile over regular human insulin, have been shown possibly because of an additional glucose control improvement with no increase or decrease in the number of SH episodes in patients with type 1 diabetes, showing similar or lower diabetes-related and total medical costs as a result of fewer inpatient hospital expenditures (16–18).

Lifetime costs of type 1 diabetes in Spain have been estimated as €97,000 (updated to 2005) per patient with an average lifespan of 59.6 years (19), giving a cost per year of €1379. Many similar studies have included a large indirect cost approximately equal to the direct costs (20) and several have indicated lower costs including an estimate of €682/patient/year in Spain (21). In our study, we have found a median rate of two episodes of SH in a 2-year period, similar to other studies such as of 1.3 episodes/patient-year, reported in a Danish–British multicentre survey of 1076 adult patients with clinical type 1 diabetes (22). Taking this rate into consideration one episode per year at a cost of €366, would be significant in relation to the above total costs of diabetes.

Although it is largely for decision makers to determine whether any extra total costs associated with more modern treatments, which reduce the incidence of SH episodes, are ‘worthwhile’ in that total benefits (financial and non-financial) exceed total treatment cost, our data would argue in favour as shown in this manuscript. For example, Holleman et al. (9) noted that the number of SH episodes which resulted in loss of consciousness in his study was reduced by the use of insulin lispro by 13 episodes (from 16 to three episodes, p = 0.004). With the 30% reduction with insulin lispro in the incidence of SH episodes overall (i.e. not just those resulting in loss of consciousness), a proportionate reduction in the number of episodes which result in the loss of consciousness, would lead to a reduction in these cases by four to five. Using the figures of Anderson (where approximately 35% of all cases who were unable to self-treat resulted in loss of consciousness) a reduction in the number of loss of consciousness cases of four or five per 100 patients per year from using insulin lispro would cost approximately €1464–1830 based on our estimate of €366 as the average total cost per SH episode. However, our estimation still may be conservative regarding real total cost, as there are circumstances after the episode of SH in daily life such us non-labour time that have not been estimated.

Data from previous economic evaluations outlining the costs of diabetic complications and associated risk factors in Spain were readily available (23–25). The estimated average cost of treating a SH episode in our study was higher than the reported annual costs for treating eye disease (€177 for laser treatment) (23), and similar to treating hyperlipidaemia (€370 for statins treatment) (24) or gangrene (€484) (25). In terms of costs, treating SH is as relevant as treating eye disease or gangrene, two very common diabetic complications.

In addition to costs, there are other negative effects of this major acute complication of insulin therapy which should be considered, for example, impaired performance in critical activities like driving can occur in all hypoglycaemic ranges leading to disturbances and practical problems in daily life (26). Additional effects, such as fear and worsened control and lower patient's quality of life and family impact have been reported (27,28).

One possible limitation of the study may be that we only reviewed data from three centres (Madrid, Barcelona and Gerona). We considered that there are no significant geographical differences in the treatment of SH in Spain.

We conclude that the use of new therapies such us insulin lispro may be associated with reductions in annual costs because of decreased number of SH and, possibly, the overall effect may be cost neutral or cost saving when total costs are considered. The costs of SH episodes should be included in the analysis of total socio-economic burden of diabetes and cost-effectiveness analyses.
